# Draft Genome Sequence of *Stenotrophomonas maltophilia* CBF10-1, an Organophosphate-Degrading Bacterium Isolated from Ranch Soil in Fairchilds, Texas

**DOI:** 10.1128/genomeA.00378-16

**Published:** 2016-05-12

**Authors:** Rupa Iyer, Ashish Damania

**Affiliations:** a Center for Life Sciences Technology, College of Technology, University of Houston, Houston, Texas, USA; b Department of Pediatrics-Tropical Medicine, Baylor College of Medicine, Houston, Texas, USA

## Abstract

*Stenotrophomonas maltophilia* CBF10-1 was isolated from a ranch in Fairchilds, Texas, USA. Its genome reveals a highly adaptable microorganism with a large complement of antibiotic and heavy metal resistance genes, efflux pumps, multidrug transporters, and xenobiotic degradation pathways.

## GENOME ANNOUNCEMENT

*Stenotrophomonas maltophilia* is a Gram-negative bacterium predominantly found in aquatic or humid environments, as well as in hospitals, where it a common bacterial contaminant of medical devices and equipment (1). A recent study shows that 4.3% of almost 75,000 Gram-negative infections were caused by *S. maltophilia* in intensive care units in the United States, with infection most often occurring via catheter or ventilator (2). Here, we report a draft genome sequence of a new strain of *S. maltophilia*, isolated from ranch soil through an Environmental Sampling Research Module undertaken by University of Houston biotechnology undergraduates (3). Designated CBF10-1, this strain of *S. maltophilia* is closely related to *S. maltophilia* K279a (2), with a battery of β-lactam antibiotic resistance genes present in addition to resistance to fluoroquinolones. Its large metallo-β-lactamase complement includes a putative methyl parathion hydrolase with approximately 58% sequence similarity to the model enzyme found in *Pseudomonas* sp. WBC-3 and *Plesiomonas* sp. M6 from China, providing it some secondary phosphotriesterase activity against organophosphate insecticides (4, 5). 

The genome sequencing of CBF10-1 was performed through Illumina MiSeq paired-end sequencing with a final sequencing coverage of 644.00×. Sequence reads were checked for quality using FastQC (6) and filtered using BBTools (7). Paired-end reads were then assembled into a total of 115 contigs with the Spades version 3.6.2 program (8). Preliminary reference-based annotation using Patric (9) was carried out to identify conserved pathways. Final *de novo* annotation was performed with Prokka (10) and the NCBI Prokaryotic Genome Automatic Annotation Pipeline (http://www.ncbi.nlm.nih.gov/genome/annotation_prok). The metabolic pathways of aromatic and heterocyclic compounds were examined through KEGG databases (11). This draft genome of strain CBF10-1 consists of a total of 4,556,616 bp encoding for 4,018 putative coding sequences, of which 36 have been classified as pseudogenes, 1,093 as hypothetical proteins, and 2,925 predicted to form known functional proteins. The genome has a high GC content of 66.49 and contains 7 rRNA, 71 tRNA, and 4 ncRNA loci.

### Nucleotide sequence accession numbers.

The *Stenotrophomonas maltophilia* CBF10-1 whole-genome shotgun project has the project accession number LTAC00000000. This version of the project (01) has the accession number LTAC01000000 and consists of sequences LTAC01000001 to LTAC010000115.
